# Single-lesion sporotrichosis triggering Sweet’s syndrome^⋆^

**DOI:** 10.1016/j.abd.2024.03.001

**Published:** 2024-06-08

**Authors:** Hiram Larangeira de Almeida, Augusto Scott da Rocha, Lilian Müller, Ana Letícia Boff

**Affiliations:** a Postgraduation in Health and Behavior, Universidade Católica de Pelotas, Pelotas, RS, Brazil; b Department of Dermatology, Universidade Federal de Pelotas, Pelotas, RS, Brazil; c Private Veterinary Clinic, Pelotas, RS, Brazil.; d Santa Casa de Misericórdia de Porto Alegre, Porto Alegre, RS, Brazil

Dear Editor,

Sporotrichosis is a deep mycosis caused by dimorphic fungi of the genus *Sporothrix*, which can be subacute or chronic, and is usually subcutaneous but may be systemic in rare cases. It can be classified into four categories: cutaneous-lymphatic (around 75% of cases), localized cutaneous (20%), disseminated and extracutaneous.^1^, ^2^

Skin lesions typically appear at the inoculation site, as an implantation mycosis, mainly when handling plants and infected soil or after trauma by infected animals.^1^, ^2^

It begins as an inflammatory papule, which develops into a nodule or gumma that becomes an ulcer and later, in cutaneous-lymphatic presentations, papules and nodules appear along one or more lymphatic cords close to the initial lesion.

In the last two to three decades in Brazil, sporotrichosis has spread zoonotically through the species *Sporothrix brasiliensis*, which is the species most often found in domestic cats - currently the main vectors of the disease in Brazil.^1^, ^2^ *Sporothrix brasiliensis* is associated with hypersensitivity reactions and atypical manifestations of sporotrichosis, such as arthritis, erythema nodosum, erythema multiforme and Sweet’s syndrome.^1^, ^2^, ^3^

Sweet’s syndrome is a rare reactional dermatosis that typically presents with symmetric painful skin lesions such as papules, nodules or erythematous/erythematous-violaceous plaques, which are often called pseudo-vesicles, due to the visual appearance on inspection. However, on palpation, they have a papular consistency. It is also called acute febrile neutrophilic dermatosis, as the presence of fever and neutrophilia is usual; histopathologically, it always presents a dense neutrophilic inflammatory infiltrate in the papillary dermis.^4^

A 51-year-old female patient who had contact with a domestic cat that was euthanized due to sporotrichosis was evaluated. The patient stated that she took care of the cat but was never scratched or bitten by it.

Two weeks after the cat had died, she developed a pustule on her thigh (Fig. 1A), treated with topical neomycin, which developed into an ecthymoid lesion (Fig. 1B). A few days later she started to develop disseminated lesions, with a pseudo-vesicular appearance (Fig. 2) accompanied by arthralgia, which made walking very difficult. With the suspicion of Sweet’s syndrome associated with sporotrichosis, given the history of exposure to the cat and no recent use of medications, oral itraconazole 100 mg/day and prednisone 40 mg/day were started. A culture was collected from the ecthymoid lesion, which was positive for *Sporothrix* spp. (Fig. 1C) and an inflammatory lesion on the back was biopsied. On histopathology there was marked edema in the superficial dermis associated with a lymphocytic and neutrophilic infiltrate and a extravasation of red blood cells. There was no spongiosis or vacuolar damage (Figure 3, Figure 4).

**Figure 1:**
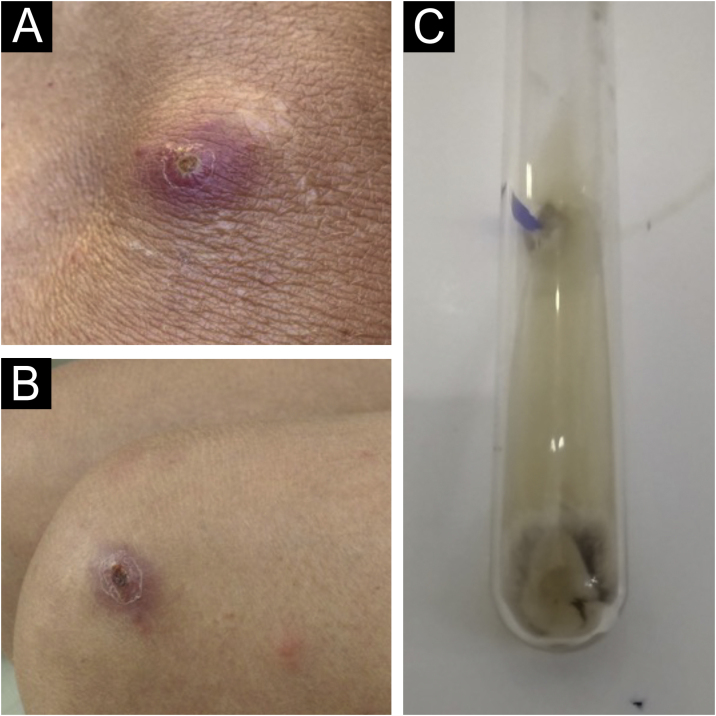
(A) Initial clinical appearance with pustule. (B) Evolution to ecthymoid lesion. (C) Positive culture for *Sporothrix* spp.

**Figure 2:**
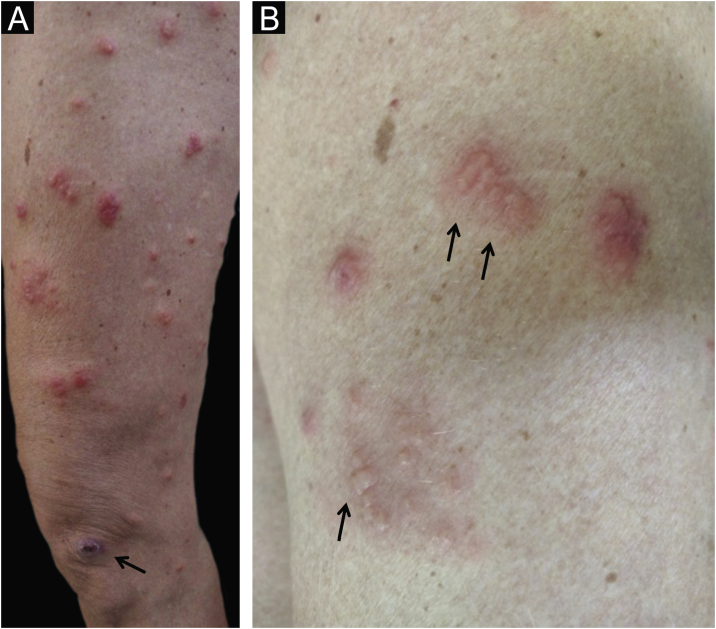
(A) Disseminated erythematous lesions; observe the ecthymoid lesion at the bottom (arrow). (B) Detail of the pseudo-vesicular appearance of the lesions (arrows).

**Figure 3:**
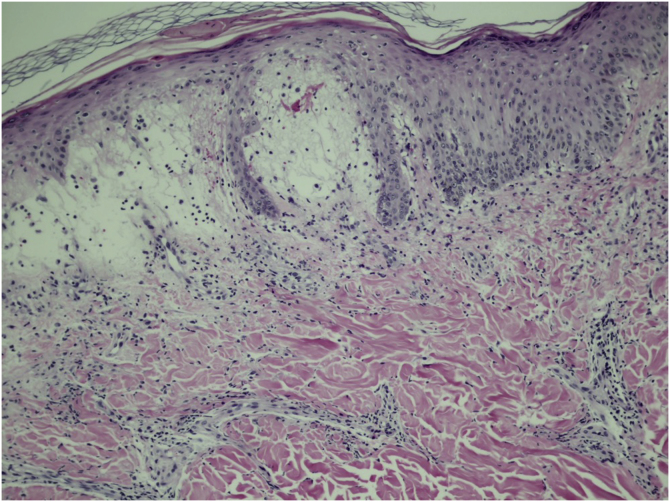
On histopathology there is marked edema of the superficial dermis associated with a lymphocytic and neutrophilic infiltrate and extravasation of red blood cells. Absence of spongiosis, vasculitis or vacuolar damage (Hematoxylin & eosin, ×100).

**Figure 4:**
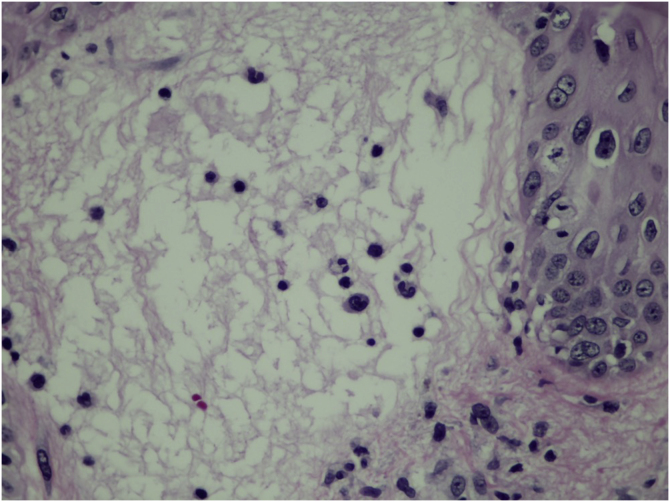
Detail of the edema of the papillary dermis, corresponding to the clinical pseudo-vesicle (Hematoxylin & eosin, ×400).

The patient experienced rapid regression of the pain and inflammatory lesions, so corticosteroids were withdrawn within seven days, and antifungal treatment was continued for 90 days, with complete resolution.

This case documents well the intense hypersensitivity reaction in a localized case of single-lesion sporotrichosis. In a series of ten cases associated with Sweet’s syndrome,^3^ nine were localized forms, as in the case described herein. The association of this hypersensitivity reaction with other deep mycoses is also well established, such as in histoplasmosis,^5^ coccidioidomycosis and also mycobacterioses.^6^

This case is peculiar due to the intense reaction associated with the oligosymptomatic form of sporotrichosis. Another interesting aspect in this case is the initial pustular lesion, probably without inoculation trauma.

## Financial support

None declared.

## Authors’ contributions

Hiram Larangeira de Almeida Jr: Approval of the final version of the manuscript; design and planning of the study; drafting and editing of the manuscript; collection, analysis and interpretation of data; effective participation in research orientation; intellectual participation in the propaedeutic and/or therapeutic conduct of the studied cases; critical review of the literature; critical review of the manuscript.

Augusto Scott da Rocha: Approval of the final version of the manuscript; design and planning of the study; drafting and editing of the manuscript; collection, analysis and interpretation of data; intellectual participation in the propaedeutic and/or therapeutic conduct of the studied cases; critical review of the literature; critical review of the manuscript.

Lilian Müller: Approval of the final version of the manuscript; design and planning of the study; drafting and editing of the manuscript; collection, analysis and interpretation of data; intellectual participation in the propaedeutic and/or therapeutic conduct of the studied cases; critical review of the literature; critical review of the manuscript.

Ana Letícia Boff: Approval of the final version of the manuscript; design and planning of the study; drafting and editing of the manuscript; collection, analysis and interpretation of data; intellectual participation in the propaedeutic and/or therapeutic conduct of the studied cases; critical review of the literature; critical review of the manuscript.

## Conflicts of interest

None declared.
